# Social and ecological disparities in anaemia among adolescent girls 15–19 years old in Nepal

**DOI:** 10.1017/S1368980023002379

**Published:** 2023-12

**Authors:** Anjana Rai, Mei Ting Chan, Smita Nambiar

**Affiliations:** 1 School of Public Health and Social Work, Queensland University of Technology, Brisbane, QLD 4059, Australia; 2 School of Exercise and Nutrition Sciences, Queensland University of Technology, Brisbane, QLD 4059, Australia

**Keywords:** Adolescent girls, Anaemia, Social determinants, Nepal

## Abstract

**Objective:**

Adolescent girls are at risk of anaemia due to increased nutrient demands because of growth, menstrual blood loss and possible pregnancies. Sociocultural and household conditions influence their anaemia risk. We aimed to identify the sociocultural and economic factors associated with anaemia among adolescent girls in Nepal.

**Design:**

The Nepal Demographic and Health Surveys (NDHS) conducted in 2006, 2011 and 2016 were pooled for secondary analysis. We used data on haemoglobin measurements for anaemia and conducted bivariate and multivariable logistic regression analyses to identify factors associated with anaemia.

**Setting:**

Nationally representative NDHS households with adolescent girls 15–19 years of age.

**Participants:**

Non-pregnant adolescent girls 15–19 years, with a haemoglobin measurement (*n =* 3731).

**Results:**

The overall prevalence of anaemia among adolescent girls was 39·6 %. Adolescents from socially disadvantaged caste/ethnicity groups were 1·42 times (95 % CI: 1·13, 1·78) more likely to have anaemia compared with those from *Brahmin/Chhetri* households. We found a counter-intuitive association between socio-economic status and anaemia where adolescents from the middle (adjusted OR (aOR) 1·37, 95 % CI: 1·01, 1·85) and highest (aOR 1·74, 95 % CI: 1·18, 2·56) quintiles were at increased odds of anaemia. Relative geographical inequality was observed where adolescents from the Terai region had 3·5 times (95 % CI: 2·32, 5·33) higher odds of anaemia.

**Conclusions:**

The disparities in the distribution of anaemia among adolescents by caste/ethnicity groups, wealth quintiles and geographical regions are evident. Reducing the anaemia burden will require addressing the social determinants of anaemia by allocating resources and expanding anaemia prevention programmes to target adolescents at higher risk.

Adolescence is a crucial developmental stage in the human lifecycle, characterised by rapid growth, sexual maturation^(1)^ and transition in autonomy^(2)^. An increased need for a range of nutrients, such as iron, accompanies these changes associated with growth and maturation^(1)^. Iron requirements among adolescent females sharply increase due to growth, menstrual blood loss and possible pregnancies^(3)^. The inability to meet increased iron requirements often leaves adolescent females vulnerable and more susceptible to anaemia^(4,5)^. Anaemia is a condition with inadequate red blood cells in the body, or the concentration of haemoglobin (Hb) falls below the cut-off point, consequently impairing oxygen transportation in the body^(6)^.

Evidence has shown anaemia to be associated with adverse consequences. In adolescents, anaemia is associated with compromised growth^(7)^, decreased cognitive function and depressed immune function^(8,9)^. A recent study in India reported that persistent anaemia in adolescents was associated with poor learning outcomes^(10)^. In adulthood, anaemia can impact productivity and overall quality of life^(11)^. Furthermore, the consequences of anaemia in adolescent mothers could lead to adverse pregnancy and intergenerational health outcomes in newborns and children^(12)^.

In South Asia, 54·6 % of adolescent girls were anaemic in 2017, and the reduction in anaemia prevalence has been slow^(1)^. In Nepal, a quarter of the population are adolescents^(13)^, and the anaemia prevalence is between 20·6 %^(14)^ and 31 %. Early marriage is prevalent, and the mean age at marriage (17·9 years) coincides with adolescence,^(13)^ which may increase the risk of anaemia^(9)^.

The causes of anaemia are multi-factorial, and a complex relationship exists with the sociocultural and economic context of where adolescents live and grow. A review on adolescent health identified income and education as vital social determinants^(15)^. Underlying risk factors such as inequalities that underscore suboptimal dietary intake^(16)^ due to food taboos^(17)^, gendered food allocation^(18)^, and low autonomy^(19)^ have been associated with the risk of anaemia. Sanitation^(20)^, poverty and disadvantaged minorities should be considered when analysing factors associated with anaemia. Another review on undernutrition among adolescents in low- and middle-income countries^(9)^ highlighted the importance of social determinants such as age, education, religion, ethnicity, working status and economic status^(3,9,21)^. They also highlight study gaps in agency and decision-making among adolescents, which might influence their nutritional status. Additionally, the WHO framework for action in accelerating the reduction of anaemia reaffirms the importance of addressing the social determinants of health and the broader context of promoting women’s and girls’ empowerment in addressing anaemia^(22)^.

This study aimed to further our knowledge of the health of older adolescent girls aged 15–19 years from Nepal. We aimed to investigate the underlying social, cultural and economic factors of anaemia. We used Madjdian et al.’s 2018^(9)^ framework to identify sociocultural and economic factors associated with anaemia among adolescent girls. Their framework on adolescent undernutrition is based on low- and lower-middle-income countries, emphasises the significance of sociocultural context and enables identification of factors at the larger community and society level, as well as the household and individual level factors.^(9)^ Additionally, we examined agency among adolescent girls in decision-making on health care, large household purchases, household purchases for daily needs and visits to family or relatives and its association with anaemia. While decision-making and empowerment are widely investigated in relation to adult women’s nutritional outcomes, decision-making agency and anaemia among adolescents have been rarely investigated.

## Methods

### Study design

We utilised the National Demographic Health Survey (NDHS) pooled datasets collected in 2006 (collected between February and August)^(23)^, 2011 (collected between January to June)^(24)^ and 2016 (collected between June 2016 to January 2017)^(13)^ and conducted a secondary data analysis. The NDHS is a nationally representative survey conducted every 5 years, documenting the nutritional status; social and other clinical information of the population in Nepal.

The NDHS used a multistage cluster sampling approach. Probability proportional to size sampling was used to select the primary sampling units (clusters), which served as the sampling frame in the second stage. Subsequently, thirty households per cluster were selected from the sampling frame. Details on survey design are provided in the NDHS reports^(13)^. We merged the women’s dataset with the household dataset using the cluster, household and respondent identifiers for each NDHS year, and we pooled the merged datasets into one. Ethical approval of the NDHS surveys (including the approval for Hb testing) was provided by the Nepal Health Research Council and the human research ethics committee at ICF and Macro International.

### Study population

This study population was adolescent girls between 15 and 19 years of age, de jure residents of households sampled in NDHS. We included all married and unmarried adolescent girls with Hb measurements available in the NDHS surveys. Data from 2006, 2011 and 2016 surveys were pooled for the analyses. As the objective of this study was to identify the factors of anaemia and the scope was not to assess the effect of any exposure, the sample size for this study was determined by the availability of Hb measurements in the dataset. A total of 3731 non-pregnant adolescent girls (*n* 975 in 2006; *n* 998 in 2011; *n* 1758 in 2016) formed the data analysis sample.

### Outcome variable

The outcome variable was anaemia (*v*. no-anaemia) in adolescent girls 15–19 years old. We used Hb concentrations adjusted for altitude and smoking, available in the NHDS datasets. In the NDHS, Hb concentrations were measured using HemoCue (Hb 201 Photometer) system in all three rounds. Capillary blood samples were taken from a finger prick and collected in a microcuvette. Hb concentrations were analysed onsite using a portable HemoCue analyser^(13,23,24)^. We used the WHO Hb cut-offs of ≥ 120 g/l for non-anaemia and < 120 g/l to categorise anaemia.^(25)^

### Possible factors

Madjdian et al.’s 2018^(9)^ framework and data available in the NDHS guided the selection of possible factors of anaemia from the dataset. Age, marital status, education, employment and caste/ethnicity were the individual socio-demographic characteristics used in this study. We calculated the age of adolescent girls from the date of the interview and their date of birth. Current marital status: unmarried *v*. married was, respectively, coded 0 and 1. Education was categorised as ‘no formal education’ if they had never been to school or never completed any formal school grade, as ‘primary level’ if they received education between grades 1 to 5 and ‘secondary and higher’ if they had grade 6 and higher qualification. We categorised employment into ‘working’ or ‘not working’ by using information on employment in the last 12 months. Caste/ethnicity was re-categorised into *Brahmin & Chhetri* (relatively advantaged), socially disadvantaged groups and other castes/ethnicity. Socially disadvantaged groups included disadvantaged *Dalit*, minority Muslim and indigenous *janajati* ethnicity, and disadvantaged others included other Terai castes, *Newars* and castes categorised as ‘others’ in the NDHS dataset.

We also explored the agency of adolescent girls in decision-making on their health care, large household purchases, household purchases for daily needs and visits to family or relatives. If decisions were made by adolescent girls autonomously or jointly, we coded it 1, else coded 0, following the survey-based women’s empowerment index method^(26)^. Individual health-related variables included BMI-for-age z scores. BMI-for-age z-scores were calculated using the Stata ‘zanthro’ command^(27)^ and then categorised into thin (< −2 sd), normal (–2 sd to +2 sd) and overweight or obese (> +2 sd) using the WHO Growth Reference charts for adolescents^(28)^.

We included household-level variables such as access to an improved source of drinking water, access to improved toilets and household wealth quintiles. Access to an improved source of water and toilet was categorised following the NDHS categories^(13)^. Drinking water from a household connection (piped), public tap or standpipe, tube well or borehole, protected dug well, protected spring, or rainwater collection and bottled water was categorised as an improved source of drinking water. For access to an improved toilet, we categorised flush or pour-flush toilet to a piped water system, septic tank or pit latrine; ventilated improved pit latrine; pit latrine with a slab or composting toilet as an improved toilet, while open-pit latrine and open defecation were categorised as no access to an improved toilet^(13)^. We used household wealth quintiles available in the NDHS, which were calculated using o on ownership of assets and housing characteristics. Further details on the calculation of wealth quintiles are provided in the NDHS reports. At the community level, we used information on residential areas classified as urban or rural and ecological zones (Mountains, Hill, and Terai).

### Statistical analysis

We performed statistical analyses on the weighted sample using sampling weights available in the NDHS datasets. We adjusted all analyses for sampling weights using the STATA ‘svy’ prefix command. All data were processed, cleaned and analysed in Stata/s e 17·0. We conducted descriptive analyses for pooled datasets to summarise adolescent girls’ socio-demographic profiles and calculate anaemia prevalence for each survey year. Descriptive mean and percentages were used for continuous and binary/categorical variables. Two-way tabulations were performed for the distribution of anaemia by possible factors.

We performed bivariate logistic regression analyses to assess the unadjusted association of possible factors with anaemia status. Variables with *P* < 0·2 in the unadjusted regression analysis were considered for inclusion in the multivariable model. We added marital status (*P* > 0·2) to assess if its association with anaemia changes in the multivariable regression. We used a backward manual stepwise regression technique for the logistic multivariable regression model where variables with larger *P* values were simultaneously removed. We removed decision-making variables on household purchases for daily needs, visiting family and friends and residential location during stepwise regression, but all other variables were retained in the model. Multicollinearity checks were also performed by assessing changes in OR estimates by > 10 % and *P*-values when variables were removed in stepwise regression. The final model was adjusted for years of survey and months of data collection (in the Nepali *sambat* calendar) to adjust for their influence on anaemia. Removal of variables with *P* > 0·05 did not change the association of remaining variables with anaemia; therefore, those variables were removed from the final model. The results of the multivariable logistic regression are reported as aORs with 95 % CI and *P* < 0·05 were considered statistically significant. We used Akaike Information Criterion, Bayesian Information Criterion and Hosmer–Lemeshow test to assess model fit.

## Results

### Prevalence of anaemia

The pooled prevalence of anaemia was 39·6 %. The distribution of anaemia prevalence in the pooled sample by possible factors is presented in Table 1. Inequality in anaemia prevalence was observed with higher anaemia prevalence among adolescents who were not employed (41·2 %), with no formal school education (46·3 %) and from socially disadvantaged caste/ethnicity (44·8 %). A slight difference was observed between never-married and married girls (39·4 % *v*. 40·8 %). Similarly, adolescents not involved in decision-making around health care (39·4 %), household purchases for daily needs (39·1 %) and visiting friends and family (39·8 %) had a higher prevalence compared with those involved in decision-making.

**Table 1:** Characteristics of adolescent girls 15–19 years old by anaemia status (*n* 3731)

Variables	Categories	Total no	Anaemia prevalence
			%
Anaemia	Present	1396	39·6
Age (mean)		3731	16·9 years
Employment	Not working	1254	41·2
	Working	2477	38·8
Education	No education	395	46·3
	Primary	735	37·0
	Secondary and higher	2601	39·2
Marital status	Never married	3042	39·4
	Married	689	40·8
BMI*	Underweight (< −2 s d)	172	44·7
	Normal weight (–2 sd to +1 sd)	3415	39·8
	Overweight/obesity (> +1 sd)	140	30·0
Decision making on health care	Girl not involved	1865	39·4
	Girl involved	223	34·8
Decision making on large household purchases	Girl not involved	1973	38·9
	Girl involved	115	39·8
Decision making on household purchases for daily needs	Girl not involved	1660	39·1
	Girl involved	98	30·6
Decision making on visiting family and friends	Girl not involved	1897	39·8
	Girl involved	191	31·2
Access to improved source of water**	No	544	32·9
	Yes	3186	40·6
Access to improved toilet	No	1579	42·9
	Yes	2152	36·9
Caste/ethnicity	Brahmin/Chhetri	1861	31·5
	Socially disadvantaged	474	44·8
	Disadvantaged others	1396	39·2
Wealth quintiles	Lowest	810	32·8
	Second	755	40·9
	Middle	698	46·1
	Fourth	788	39·5
	Highest	680	38·2
Residence	Urban	1361	38·8
	Rural	2370	39·9
Ecological zone	Mountains	499	27·5
	Hills	1557	26·6
	Terai	1675	52·6

* BMI available for *n* 3727 and *n* 1 was a flagged case in the dataset.

** *n* 1 unidentified other source, data available for *n* 3730.

Surprisingly, a higher prevalence of anaemia was observed among those with access to improved water but lower among those with access to an improved toilet. Socially disadvantaged and other caste groups had higher anaemia prevalence. Higher anaemia prevalence among adolescents in the second, middle, fourth and highest quintiles was observed (Table 1). Disparities in the distribution of anaemia were also evident in ecological zones where the Terai region bore the highest burden of anaemia. For socio-demographic characteristics of adolescent girls included in this analysis, please see online supplementary material, Supplemental Table 1.

### Factors associated with anaemia

In the bivariate analyses, age, education, BMI, caste/ethnicity, access to an improved toilet facility, wealth quintiles and ecological zones were crudely associated with anaemia. In the multivariable model adjusted for years and months of data collection, we found caste/ethnicity, wealth and ecological zone associated with anaemia (Table 2).

**Table 2:** Unadjusted and adjusted associations for factors of anaemia among adolescent girls 15–19 years old

Possible factors	Categories	Bivariate models			Multivariable model (*n* 3726)		
		cOR	95 % CI	*P*	aOR	95 % CI	*P*
Age		0·94	0·87, 1·01	0·097	0·96	0·89, 1·04	0·313
Education	No education	Ref			Ref		
	Primary	0·68	0·49, 0·96	0·026	0·89	0·63, 1·25	0·509
	Secondary or higher	0·75	0·56, 1·00	0·052	1·01	0·73, 1·42	0·932
Current marital status	Never married	Ref			Ref		
	Married	1·06	0·87, 1·30	0·557	1·06	0·84, 1·34	0·617
Employment status	Not working	Ref			Ref		
	Working	0·90	0·76, 1·08	0·273	1·08	0·86, 1·35	0·528
BMI	Normal weight (–2 sd to +1 sd)	Ref			Ref		
	Thinness (< −2 sd)	0·82	0·56, 1·20	0·305	1·01	0·68, 1·51	0·956
	Overweight/obesity (> +1 sd)	0·53	0·29, 0·98	0·043	0·71	0·45, 1·13	0·150
Decision making on health care	Girl not involved	Ref			–		
	Girl involved	0·82	0·50, 1·33	0·416	–		
Decision making on large household purchases	Girl not involved	Ref			–		
	Girl involved	1·04	0·65, 1·67	0·866	–		
Decision making on household purchases for daily needs	Girl not involved	Ref			–		
	Girl involved	0·69	0·41, 1·16	0·159	–		
Decision making on visiting family and friends	Girl not involved	Ref			–		
	Girl involved	0·69	0·45, 1·04	0·074	–		
Caste/ethnicity	Brahmin/Chhetri	Ref			Ref		
	Socially disadvantaged	1·40	1·02, 1·93	0·037	1·42	1·13, 1·78	0·003
	Disadvantaged others	1·77	1·32, 2·36	< 0·001	1·00	0·74, 1·37	0·975
Access to improved source of water	No	Ref			Ref		
	Yes	1·33	1·96, 1·85	0·089	0·89	0·65, 1·22	0·467
Access to improved toilet	No	Ref			Ref		
	Yes	0·78	0·62, 0·98	0·035	0·81	0·65, 1·00	0·053
Wealth quintile	Lowest	Ref			Ref		
	Second	1·42	1·12, 1·80	0·004	1·17	0·90, 1·53	0·237
	Middle	1·75	1·33, 2·30	< 0·001	1·37	1·01, 1·85	0·040
	Fourth	1·34	0·97, 1·85	0·08	1·29	0·91, 1·82	0·147
	Highest	1·27	0·94, 1·70	0·115	1·74	1·18, 2·56	0·005
Residential location	Urban	Ref			–		
	Rural	0·05	–0·23, 0·32	0·745	–		
Ecological zone	Mountains	Ref			Ref		
	Hill	0·96	0·66, 1·38	0·807	1·04	0·71, 1·53	0·821
	Terai	2·92	1·94, 4·41	< 0·001	3·51	2·32, 5·33	< 0·001

Model adjusted for year and months (Nepali Sambat calendar) of data collection.

Akaike Information Criterion: 4613·28, Bayesian Information Criterion: 4806·19, Hosmer–Lemeshow test *P* = 0·21 from unweighted model.

Adolescent girls from a socially disadvantaged caste/ethnicity had higher odds of anaemia than the *Brahmin/Chhetri* caste (aOR 1·42, 95 % CI: 1·13, 1·78). Surprisingly, we found adolescent girls from the middle (aOR 1·37, 95 % CI: 1·01, 1·85) and highest (aOR 1·74, 95 % CI: 1·18, 2·56) wealth quintiles were at increased odds of anaemia compared with those from the lowest wealth quintile. Additionally, we assessed the prevalence of anaemia by wealth quintile for each NDHS year. The prevalence of anaemia was 38·5 % in 2006, 38·4 % in 2011 and 42·9 % in 2016. In all years, the lowest quintile had the least prevalence of anaemia compared with higher quintiles (see online supplementary material, Supplemental Fig. 1).

Relative geographical inequality was observed in this study. Adolescent girls from the Terai region were at increased odds of anaemia, respectively, by 3·5 times (aOR 3·51, 95 % CI: 2·32, 5·33) compared with adolescent girls in the Mountain region. Anaemia was not associated with age, education, marital status, employment, BMI categories, source of drinking water and access to improved toilets in the multivariable model.

## Discussion

This study adds to the research on the sociocultural and economic factors associated with anaemia among adolescent girls 15–19 years old in Nepal. We found an unexpected counter-intuitive association between higher socio-economic status and higher odds of anaemia. Disadvantaged caste/ethnicity and Terai ecological zone were associated with higher odds of anaemia. Surprisingly, we did not find an association between household sanitation or individual factors and anaemia.

The high prevalence of anaemia in adolescent girls is a significant health problem. The population-level prevalence of anaemia was 38·5 % in 2006, 38·4 % in 2011 and 42·9 % in 2016 among adolescent girls 15–19 years old. The prevalence of anaemia in this study was higher than that reported in other Nepalese studies using nationally representative data. These studies reported a prevalence of 27–29 %^(29)^ and 37 %^(20)^ among 15–19 years old and 20·6 % among 10–19 years old^(14)^ Nepalese adolescents. Other sub-national studies report anaemia prevalence between 33 and 43 % among different adolescent age groups^(30–32)^.

We observed a counter-intuitive association between socio-economic status and anaemia. Adolescent girls from the highest wealth quintiles had higher odds of anaemia than those in the lowest. This finding contradicts the findings of other studies from India, which reported higher socio-economic status as protective of anaemia^(33,34)^. In studies by Ford et al. 2022^(14)^ and Chalise et al. 2018^(20)^, their final multivariable models did not include socio-economic status; therefore, we are unable to make comparisons. However, there is some evidence in their descriptive tables that girls from poorer households have lower anaemia prevalence than richer households which is consistent with our findings. In a study among adult women, using the same NDHS dataset, Rai et al. 2020 also reported that higher socio-economic status was associated with higher odds of anaemia among women of reproductive age in Nepal^(35)^. In their findings, anaemia was not concentrated in the poorest socio-economic groups only. We assume two reasons, first, the households in lower wealth quintiles may depend on low-cost iron-rich green leafy vegetables providing some of their daily iron needs^(36)^. Second, the wealthier quintiles have resources to access a more westernised diet that is low in iron but higher in refined carbohydrates and fat such as ultra-processed fast-foods and packaged foods. Low- and middle-income countries undergoing economic transition face a rapid increase in consumption of ultra-processed food and beverages^(37)^. Perhaps, the wealthier households may be foremost in undergoing transition from traditional to westernised diets. In a review, fast-food consumption and snacking were reported to be high in adolescents from urban areas and those attending private schools in India^(38)^. In Brazil, adolescents from families earning more than a minimum wage were likely to have a junk food consumption pattern^(39)^. However, further studies are needed to assess these assumptions and investigate the relationship of wealth and anaemia among adolescents. Similarly, understanding food choices and transitions, especially among adolescent girls, may contribute to understanding the higher anaemia burden among wealthier quintiles.

Several studies have reported that adverse sanitation conditions, such as open defecation increased the risk of infections such as worm infestations, increasing the risk of anaemia^(40)^. We did not have data on helminth infections but used proxies such as access to improved toileting facilities. It was associated with reduced odds in the bivariate analysis but not in the multivariable analysis. While the odds of anaemia were lower among those with access to improved toileting facilities, the association was insignificant (*p* > 0·05). The absence of association between access to the toilet^(20)^ and open defecation (among girls)^(14)^ with anaemia was reported in studies among adolescents from Nepal. Future research may also need to explore community sanitation coverage and the use of updated sanitation measures, such as safely managed water, sanitation and hygiene^(41)^.

In the unadjusted model, we found adolescents from socially disadvantaged and disadvantaged other groups were at significantly increased risk of anaemia. In the multivariable model, only a socially disadvantaged group was associated with a higher risk of anaemia. While Ford et al., 2020 have used a different categorisation of ethnicity, the finding in our study is similar to their association between *Janajati* ethnicity and higher odds of anaemia^(14)^. We speculate a combination of disadvantages, such as barriers in health service utilisation^(42)^, limited access to education and cultural dietary practices^(9,14)^, intersect to determine poor health outcomes in socially disadvantaged groups. *Janajati* ethnicity of the hills constituted > 60 % of the socially disadvantaged group; they have limited access to arable land and agricultural resources resulting in food insecurity. Evidence shows that improving food security through food and agricultural interventions improves nutritional outcomes in South Asia^(43)^. Even though research and development programmes use caste and ethnicity in assessing poor health outcomes^(42)^ and as an indicator of social inclusion^(44)^, understanding the complex mechanism that underlies the association of caste/ethnicity with poor health outcomes, including anaemia, is under-researched. Quantitative pathway analysis was beyond our scope because of the lack of required information. Intersectional research methods which consider the intersections of other social systems with caste/ethnicity may provide an in-depth understanding of the association observed in this study^(45)^.

Adolescent girls in the Terai region were at increased risk of anaemia. Terai is the lowlands of Nepal, which has been evidenced to have a higher risk of anaemia^(20)^. The increased risk of anaemia in lowlands may be attributed to a higher incidence of hookworm infestation and malaria^(40)^. Nutritional deprivation may be exacerbated by gendered bias in household food allocation^(18)^, food norms and taboos during menstruation^(17,29)^ and low autonomy in food choices and consumption in the adolescent population^(19)^. Sub-national investigation of anaemia and its factors will benefit the design of effective anaemia prevention programmes.

We did not find a significant (*p* < 0·05) association between household decision-making and anaemia in the unadjusted models; however, adolescents involved in decision-making on health care, household purchases for daily needs and visiting family and friends had lower prevalence and odds of anaemia, suggesting some possible benefits of higher agency. Previous study on agency among adolescent girls in Africa has also reported a lack of association between decision-making agency on Hb and anaemia^(46)^. Further studies are needed to confirm the findings of agency and anaemia observed in our study.

This study contributes to the limited evidence base in the adolescent girl population. Findings from this analysis suggest targeting adolescent subgroups with higher anaemia burdens from socially disadvantaged caste/ethnicity, wealthier households and the Terai region. Reducing the anaemia burden might not be possible by only targeting the immediate factors. Addressing the social determinants of anaemia is imperative. Furthermore, narrowing the disparities observed will require additional multi-sectoral support.

Current iron-folic acid supplementation programmes through schools may benefit from incorporating more comprehensive interventions targeting social and economic factors. Targeting anaemia prevention for adolescent girls via schools may still be an effective way to reach most girls who spend a substantial amount of their day in school. However, adolescents who have dropped out of school and those who are newly married are hard to reach. Iron-folic acid supplementation at the primary health care level, currently targeted for pregnant women, should expand their services to include these hard-to-reach adolescents. For Terai regions, supplementation with benefit from regular tests for malaria reduction campaigns to prevent anaemia-malaria co-infection^(47)^ and continuing to address open defecation and sanitation concerns. Fortified foods have shown benefits in reducing iron deficiency among adolescents in India^(48)^ and Bangladesh^(49)^. The Government of Nepal has identified food fortification as a strategy to increase the consumption of nutrient-rich foods^(50)^, which was acceptable in remote Nepali food-insecure populations^(51)^. Future studies should investigate the potential of fortified foods in reducing the anaemia burden among adolescents in Nepal.

This study has several strengths. First, our study reaffirms that adolescent females are a vulnerable group needing immediate anaemia intervention. Second, one of the strengths of our findings is the association between wealth and anaemia, which highlights that wealth does not equate to better anaemia outcomes, and all groups should be the focus of nutrition interventions. Future research should look specifically into pathways of wealth and anaemia, as there may be different determinants in different socio-economic groups. Another interesting finding of our analysis is the investigation of agency and anaemia. Our descriptive results indicate that girls have limited agency on fundamental human rights such as healthcare, household decisions and relationships. While our results did not find an association between agency and anaemia, it does not imply evidence of no association. Future studies could use path analysis or indices to assess nutrition empowerment^(52)^ and its association with anaemia.

This study has limitations. We could not include other critical direct causes of anaemia, including malaria, parasitic infections, helminth, haemoglobinopathies and other inflammatory diseases, as these were not available in the data source. Additionally, dietary diversity information was limited to adolescent girl mothers with a live birth in the five years preceding the survey of 2006 and 2016. Food security-related questions were only available in NDHS 2011 and 2016; only seven of nine standard household food insecurity access scale questions were included in the 2011 NDHS. Therefore, dietary diversity and food security questions were not included in the analysis because of a very small sample when models were executed. In our sample of non-pregnant adolescent girls, some had a pregnancy in the past, which may also have carry-over effects on anaemia. DHS datasets are widely used in Nepal and other low- and middle-income countries for research and programme planning. We suggest that future DHS surveys expand their current measurement of dietary diversity, food security and iron-folic acid consumption to all adolescent girls which are currently limited to adolescents with specific characteristics. Due to the nature of this analysis, we cannot infer the causality of studied factors with anaemia.

### Conclusion

This study reaffirms that anaemia is a persisting problem among adolescent girls. We identified disparities in anaemia prevalence among wealth, caste/ethnicity groups and ecological zones. Reducing the anaemia burden will require addressing the social and economic determinants of anaemia. Programmes have overlooked the adolescent girl population, but there is an urgent need for investment in nutrition interventions for adolescent girls to meet the Global Nutrition Targets 2025 and Sustainable Development Goals 2030.

## Supporting information

Rai et al. supplementary material 1 Rai et al. supplementary material

Rai et al. supplementary material 2 Rai et al. supplementary material

## Supplementary material

For supplementary material accompanying this paper visit https://doi.org/10.1017/S1368980023002379
