# Supplementary figures and images for: Social and ecological disparities in anaemia among adolescent girls 15–19 years old in Nepal
Source: Public Health Nutr. 2023 Oct 31;26(12):2973–81. doi: 10.1017/S1368980023002379 (PMC10755426; doi:10.1017/S1368980023002379)

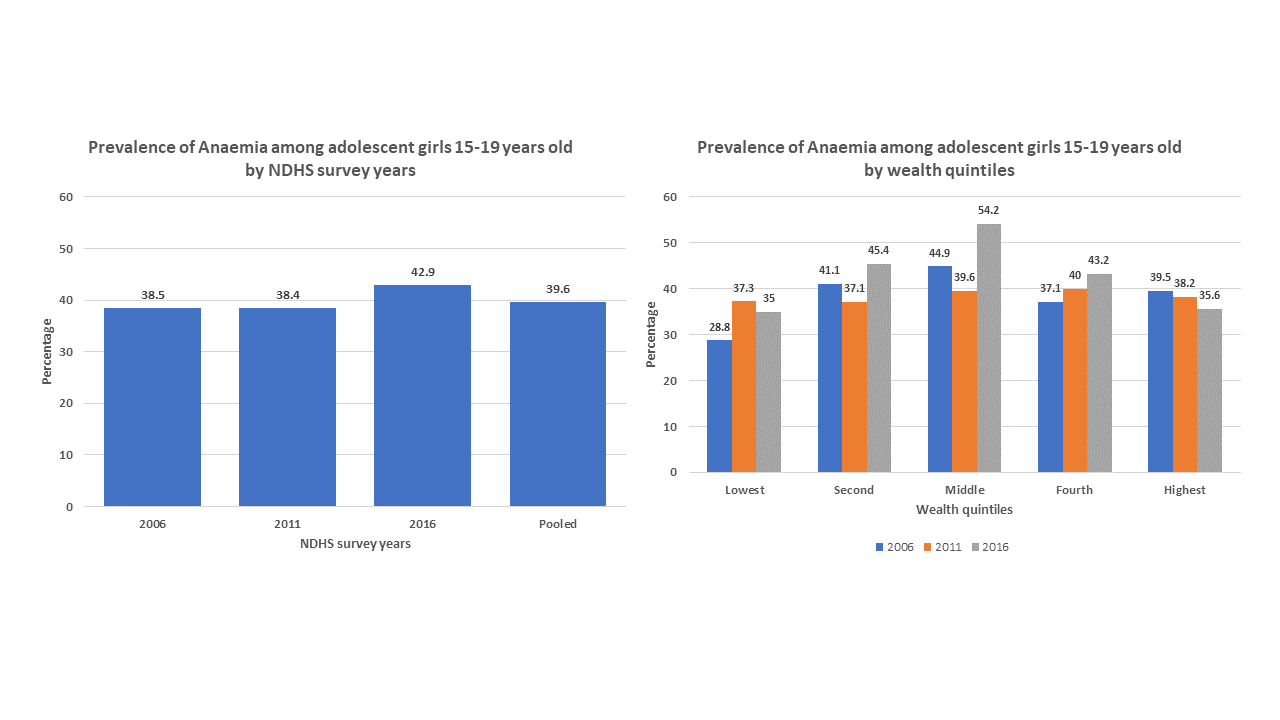

Supplement: Rai et al. supplementary material 1 — Rai et al. supplementary material [file S1368980023002379sup001.zip › Supplementary figure1_PHN.png]
