# Supplementary material for: Social and ecological disparities in anaemia among adolescent girls 15–19 years old in Nepal
Source: Public Health Nutr. 2023 Oct 31;26(12):2973–81. doi: 10.1017/S1368980023002379 (PMC10755426; doi:10.1017/S1368980023002379)
Supplement: Rai et al. supplementary material 2 — Rai et al. supplementary material [file S1368980023002379sup002.docx]

Supplementary Table 1: Characteristics of adolescent girls 15–19 years old

| **Variables** | **Categories** | **Total** | |
| --- | --- | --- | --- |
|  |  | **%** | **N** |
| Age (mean) |  | 16.9 years | 3731 |
| Employment | Not working | 35.3 | 1254 |
|  | Paid employment | 64.7 | 2477 |
| Education | No education | 12.0 | 395 |
|  | Primary | 19.9 | 735 |
|  | Secondary and higher | 68.1 | 2601 |
| Marital status | Never married | 81.8 | 3042 |
|  | Married | 18.2 | 689 |
| Visited health facility in the last 12 months | No | 65.1 | 2387 |
|  | Yes | 34.9 | 1344 |
| Contraceptive use | No | 95.6 | 3566 |
|  | Yes | 4.4 | 165 |
| BMI* | Underweight (<-2 SD) | 5.1 | 172 |
|  | Normal weight (-2 SD to +1 SD) | 91.1 | 3415 |
|  | Overweight/obesity (>+1 SD) | 3.8 | 140 |
| Decision-making on healthcare | Girl not involved | 89.2 | 1865 |
|  | Girl involved | 10.8 | 223 |
| Decision-making on large household purchases | Girl not involved | 93.6 | 1973 |
|  | Girl involved | 6.4 | 115 |
| Decision-making on household purchases for daily needs | Girl not involved | 93.4 | 1660 |
|  | Girl involved | 6.6 | 98 |
| Decision-making on visiting family and friends | Girl not involved | 90.4 | 1897 |
|  | Girl involved | 9.6 | 191 |
| Access to an improved source of water** | No | 12.3 | 544 |
|  | Yes | 87.7 | 3186 |
| Access to improved toilet facilities | No | 45.0 | 1579 |
|  | Yes | 55.0 | 2152 |
| Caste/ethnicity | Brahmin/Chhetri | 32.7 | 1861 |
|  | Socially disadvantaged | 52.2 | 474 |
|  | Disadvantaged others | 15.1 | 1396 |
| Wealth quintiles | Lowest | 19.1 | 810 |
|  | Second | 20.7 | 755 |
|  | Middle | 20.1 | 698 |
|  | Fourth | 22.3 | 788 |
|  | Highest | 17.8 | 680 |
| Residence | Urban | 26.8 | 1361 |
|  | Rural | 73.2 | 2370 |
| Anaemia | Absent | 60.4 | 2335 |
|  | Present | 39.6 | 1396 |

^*^BMI available for N= 3727 and n=1 was a flagged case in the dataset, **n=1 unidentified other source, data available for N= 3730

Supplementary Table 2: Adjusted associations for factors (including food security) of anaemia among adolescent girls 15–19 years old: Year 2011 and 2016

| **Possible factors** | **Categories** | **Multivariable model 2011 (N=997)** | | **Multivariable model 2016 (N=975)** | |
| --- | --- | --- | --- | --- | --- |
|  |  | **aOR [95% CI]** | **p** | **aOR [95% CI]** | **p** |
| Age |  | 0.98 (0.85, 1.13) | 0.792 | 0.88 (0.76, 1.02) | 0.095 |
| Education | No education | **Ref** |  | **Ref** |  |
|  | Primary | 1.39 (0.66, 2.95) | 0.386 | 0.50 (0.22, 1.15) | 0.104 |
|  | Secondary or higher | 1.22 (0.61, 2.44) | 0.579 | 0.83 (0.41, 1.67) | 0.598 |
| Current marital status | Never married | **Ref** |  | **Ref** |  |
|  | Married | 1.44 (0.92, 2.25) | 0.107 | 0.85 (0.56, 1.30) | 0.453 |
| Employment status | Not working | **Ref** |  | **Ref** |  |
|  | Working | 1.54 (1.04, 2.27) | 0.030 | 1.26 (0.92, 1.74) | 0.154 |
| BMI | Normal weight (-2 SD to +1 SD) | **Ref** |  | **Ref** |  |
|  | Thinness (<-2 SD) | 0.66 (0.25, 1.72) | 0.397 | 0.72 (0.30, 1.68) | 0.442 |
|  | Overweight/obesity (>+1 SD) | 0.79 (0.32, 1.95) | 0.605 | 0.76 (0.35, 1.66) | 0.494 |
| Caste/ethnicity | Brahmin/Chhetri | **Ref** |  | **Ref** |  |
|  | Socially disadvantaged | 1.13 (0.79, 1.63) | 0.502 | 1.31 (0.89, 1.94) | 0.167 |
|  | Disadvantaged others | 0.60 (0.28, 1.29) | 0.193 | 1.24 (0.73, 2.12) | 0.427 |
| Access to improved source of water | No | **Ref** |  | **Ref** |  |
|  | Yes | 0.82 (0.44, 1.55) | 0.542 | 0.99 (0.56, 1.76) | 0.980 |
| Access to improved toilet | No | **Ref** |  | **Ref** |  |
|  | Yes | 0.55 (0.35, 0.84) | 0.007 | 0.94 (0.58, 1.54) | 0.813 |
| Household Food Security | Food secure | **Ref** |  | **Ref** |  |
|  | Mildly food insecure | 0.89 (0.53, 1.5) | 0.661 | 1.61 (1.06, 2.46) | 0.027 |
|  | Moderately food insecure | 0.83 (0.51, 1.38) | 0.476 | 1.47 (0.95, 2.27) | 0.085 |
|  | Severely food insecure | 0.67 (0.40, 1.12) | 0.129 | 0.69 (0.33, 1.44) | 0.320 |
| Wealth quintile | Lowest | **Ref** |  | **Ref** |  |
|  | Second | 1.05 (0.64, 1.73) | 0.848 | 1.28 (0.82, 1.99) | 0.269 |
|  | Middle | 1.31 (0.70, 2.44) | 0.402 | 1.40 (0.83, 2.38) | 0.208 |
|  | Fourth | 1.63 (0.80, 3.32) | 0.180 | 1.22 (0.68, 2.18) | 0.501 |
|  | Highest | 2.75 (1.21, 6.23) | 0.016 | 1.02 (0.51, 2.03) | 0.956 |
| Ecological zone | Mountains | **Ref** |  | **Ref** |  |
|  | Hill | 1.16 (0.69, 1.95) | 0.58 | 0.62 (0.33, 1.18) | 0.148 |
|  | Terai | 3.28 (1.70, 6.34) | <0.001 | 1.84 (0.89, 3.78) | 0.098 |

Note: models adjusted for months (Nepali Sambat calendar) of data collection
